# Supplementary material for: The trust in AI-generated health advice (TAIGHA) scale and short version (TAIGHA-S): Development and validation study
Source: PLOS Digit Health. 2026 Jul 2;5(7):e0001488. doi: 10.1371/journal.pdig.0001488 (PMC13327204; doi:10.1371/journal.pdig.0001488)
Supplement: S2 Appendix — (PDF) [file pdig.0001488.s002.pdf]

Trust in AI-Generated Health Advice Short (TAIGHA-S) Scale

To what extent do you agree with the following statements?

(5-point Likert scale: Strongly Disagree, Disagree, Neutral, Agree, Strongly Agree)

Trust

I feel positive about relying on the AI's advice.

I believe the AI's advice will help me make good health decisions.

Distrust

I think following the AI's advice could pose health risks.

I feel tense about taking the AI's advice into account.
