# Supplementary material for: The trust in AI-generated health advice (TAIGHA) scale and short version (TAIGHA-S): Development and validation study
Source: PLOS Digit Health. 2026 Jul 2;5(7):e0001488. doi: 10.1371/journal.pdig.0001488 (PMC13327204; doi:10.1371/journal.pdig.0001488)
Supplement: S3 Appendix — (DOCX) [file pdig.0001488.s003.docx]

**S3 Appendix: Additional Information and Analyses**

**Textbox A. Template Used in Item Generation.**

| **^item.attributes <- list(^**  **^trust = c("cognitive trust", "affective trust"),^**  **^distrust = c("cognitive distrust", "affective distrust")^**  **^)^**  **^sub.domain <- "Trust in AI‑generated Health Advice"^**  **^scale.title <- "Trust in AI‑Generated Health Advice Scale"^**  **^system.role <- paste(^**  **^"You are an expert psychometrician writing robust, clear, 8th‑grade‑reading-level",^**  **^"items that measure only the user’s own willingness to be vulnerable (trust) or protective",^**  **^"(distrust) toward the health advice they just received from an AI. ",^**  **^"Items must refer only to the user's own",^**  **^"feelings or expected outcomes (good or bad) to the AI's advice, not to the AI's features. "^**  **^)^**  **^item.type.definitions <- list(^**  **^trust = paste(^**  **^"Trust = willingness to follow this health advice from the AI because you expect it to help you.",^**  **^"Cognitive trust: expectation that following the AI's advice will lead to positive health outcomes.",^**  **^"Affective trust: positive feelings, such as comfort, reassurance, or peace of mind when considering or relying on the AI's advice itself."^**  **^),^**  **^distrust = paste(^**  **^"Distrust = protective stance; avoiding this health advice because you expect it to harm you.",^**  **^"Cognitive distrust: expectation that following the AI's advice will lead to negative health outcomes.",^**  **^"Affective distrust: negative feelings, such as anxiety, unease, or worry, when considering or relying on the AI's advice itself."^**  **^)^**  **^)^**  **^item.examples <- c(^**  **^# Cognitive Trust^**  **^"Following the AI's advice will improve my health.",^**  **^"I expect that acting on the AI's recommendation will help me feel better.",^**  **^# Affective Trust^**  **^"I feel secure about following the AI's advice.",^**  **^"I feel reassured by the idea of relying on the AI’s recommendation.",^**  **^# Cognitive Distrust^**  **^"Following the AI's advice could make my health worse.",^**  **^"Acting on the AI's recommendation might harm me.",^**  **^# Affective Distrust^**  **^"I worry about my safety if I follow the AIs advice.",^**  **^"I feel uneasy when I consider relying on the AI’s advice."^**  **^)^** |
| --- |

**Figure B. Scree Plot for Exploratory Factor Analysis.**

**Table C. Factor Loadings in Exploratory Factor Analysis.**

| **Item** | **Trust Factor** | **Distrust Factor** |
| --- | --- | --- |
| Trust Item 1 | 0.828 |  |
| Trust Item 2 | 0.789 |  |
| Trust Item 3 | 0.825 |  |
| Trust Item 4 | 0.825 |  |
| Trust Item 5 | 0.882 |  |
| Distrust Item 1 | -0.318 | 0.576 |
| Distrust Item 2 | -0.117 | 0.722 |
| Distrust Item 3 |  | 0.862 |
| Distrust Item 4 |  | 0.936 |
| Distrust Item 5 | -0.175 | 0.754 |

**Table D. Goodness-of-Fit Indices for a One-Factor Structure and a Two-Factor Structure.**

| **Fit Index** | **One-Factor Structure** | **Two-Factor Structure** |
| --- | --- | --- |
| GFI | 1.00 | 1.00 |
| CFI | 0.89 | 0.98 |
| TLI | 0.86 | 0.98 |
| NFI | 0.88 | 0.97 |
| RMSEA | 0.17 | 0.07 |
| SRMR | 0.05 | 0.03 |

Note: In a model comparison, the two-factor model fit significantly better than the one-factor model (Δχ²(1) = 125.27, p < .001).

**Table E. Inter-Item Correlation of Trust Subscale.**

|  | **Trust Item 1** | **Trust Item 2** | **Trust Item 3** | **Trust Item 4** | **Trust Item 5** |
| --- | --- | --- | --- | --- | --- |
| Trust Item 1 | 1 |  |  |  |  |
| Trust Item 2 | 0.70 | 1 |  |  |  |
| Trust Item 3 | 0.77 | 0.72 | 1 |  |  |
| Trust Item 4 | 0.77 | 0.70 | 0.78 | 1 |  |
| Trust Item 5 | 0.76 | 0.71 | 0.77 | 0.79 | 1 |

**Table F. Inter-Item Correlation of Distrust Subscale.**

|  | **Distrust Item 1** | **Distrust Item 2** | **Distrust Item 3** | **Distrust Item 4** | **Distrust Item 5** |
| --- | --- | --- | --- | --- | --- |
| Distrust Item 1 | 1 |  |  |  |  |
| Distrust Item 2 | 0.70 | 1 |  |  |  |
| Distrust Item 3 | 0.64 | 0.70 | 1 |  |  |
| Distrust Item 4 | 0.75 | 0.73 | 0.76 | 1 |  |
| Distrust Item 5 | 0.77 | 0.71 | 0.73 | 0.81 | 1 |
